# Inverse baseline expression pattern of the NEP/neuropeptides and NFκB/proteasome pathways in androgen-dependent and androgen-independent prostate cancer cells

**DOI:** 10.1186/1475-2867-11-13

**Published:** 2011-05-15

**Authors:** Anna Patrikidou, Panagiotis J Vlachostergios, Ioannis A Voutsadakis, Eleana Hatzidaki, Rosalia-Maria Valeri, Chariklia Destouni, Effie Apostolou, Danai Daliani, Christos N Papandreou

**Affiliations:** 1 Department of Medical Oncology, University Hospital of Larissa, Larissa, Greece; 2 Department of Cytopathology, "Theagenio" Cancer Hospital, Thessaloniki, Greece; 3 Institute of Molecular Biology, Genetics and Biotechnology, Biomedical Research Foundation, Academy of Athens, Athens, Greece

## Abstract

**Background:**

Castration-resistance in prostate cancer (PC) is a critical event hallmarking a switch to a more aggressive phenotype. Neuroendocrine differentiation and upregulation of NFκB transcriptional activity are two mechanisms that have been independently linked to this process.

**Methods:**

We investigated these two pathways together using *in vitro *models of androgen-dependent (AD) and androgen-independent (AI) PC. We measured cellular levels, activity and surface expression of Neutral Endopeptidase (NEP), levels of secreted Endothelin-1 (ET-1), levels, sub-cellular localisation and DNA binding ability of NFκB, and proteasomal activity in human native PC cell lines (LnCaP and PC-3) modelling AD and AI states.

**Results:**

At baseline, AD cells were found to have high NEP expression and activity and low secreted ET-1. In contrast, they exhibited a low-level activation of the NFκB pathway associated with comparatively low 20S proteasome activity. The AI cells showed the exact mirror image, namely increased proteasomal activity resulting in a canonical pathway-mediated NFκB activation, and minimal NEP activity with increased levels of secreted ET-1.

**Conclusions:**

Our results seem to support evidence for divergent patterns of expression of the NFκB/proteasome pathway with relation to components of the NEP/neuropeptide axis in PC cells of different level of androgen dependence. NEP and ET-1 are inversely and directly related to an activated state of the NFκB/proteasome pathway, respectively. A combination therapy targeting both pathways may ultimately prove to be of benefit in clinical practice.

## Background

The 26S proteasome is part of the ubiquitin-proteasome system (UPS) and constitutes a large multiprotein complex present in all cells, both in the cytoplasm and nucleus, which degrades ubiquitinated proteins. The proteasome targets proteins that are damaged, oxidised or misfolded. Furthermore, it orchestrates the orderly degradation of regulatory proteins that govern cell cycle, transcription factor activation, apoptosis, and cell trafficking [1,2].

The activation of the nuclear factor kappa B (NFκB), a key transcription factor, is dependent on proteasome-mediated degradation of the inhibitory protein IκBα [3]. Thereby, the proteasome regulates the transcriptional activity of NFκB. NFκB induces expression of cell adhesion molecules (i.e., E-selectin, intercellular adhesion molecule-1, vascular cell adhesion molecule-1), prosurvival proteins (i.e., Bcl-2), and growth factors, like interleukin-6 (IL-6), thus promoting cell survival, angiogenesis, and metastasis, related to cancer progression and resistance to chemotherapy in various solid tumors, including PC [2,4].

Neutral endopeptidase 24.11 (NEP, neprilysin, enkephalinase, CALLA, CD10, EC 3.4.24.11) is a thermolysin-like zink metallopeptidase of the M13 family that is normally expressed by numerous tissues, including prostate. This enzyme is an integral plasma membrane ectopeptidase that plays an important role in turning off peptide signalling events at the cell surface. It cleaves peptide bonds on the amino side of hydrophobic amino acids and inactivates a variety of physiologically active peptides, including atrial natriuretic factor, substance P, bradykinin, oxytocin, Leu- and Met-enkephalins, neurotensin, endothelin, bombesin, and bombesin-like peptides, which are collectively termed neuropeptides (NPs) [5-8]. NEP reduces the local concentration of NP available for receptor binding and signal transduction via G-protein receptor coupling. It has been implicated in controlling cellular proliferation by hydrolysing NPs such as endothelin and bombesin-like peptides, found to be potent mitogens for both benign and malignant cells [9,10]. Loss or decrease in NEP expression has been reported in a variety of malignancies, including PC [11]. Reduced NEP allows an accumulation of higher peptide concentrations at the cell surface and may facilitate the development and progression of neoplasia [7,12].

The aberration of the NFκB/UPS pathway and the NEP/NPs axis have been independently linked to the development and progression of PC [2,13-17]. In this work we have hypothesised that both increased NP signalling as a result of NEP loss and overactivated NFκB-signalling emanating from increased proteasome activity are features of an evolving AD-to-AI phenotype. We therefore investigated these pathways together using in vitro models of androgen-dependence and -independence PC states.

## Methods

### Cell culture and reagents

The human prostate carcinoma lines LnCaP and PC-3 as well as the HeLa cell line were all purchased from the European Collection of Animal Cell Cultures (ECACC, Health Protection Agency, Salisbury, UK) and all experiments were performed within six months from purchase. The cell lines were cultured in RPMI 1640 (Euroclone, UK) supplemented with 10% heat-inactivated FBS (GIBCO, UK), 5% L-glutamine (GIBCO, UK) and 1% penicillin-streptomycin (Euroclone, UK) at 37°C in a humidified 5% CO_2 _atmosphere. Suc-Ala-Ala-Phe-pNa chromogenic substrate for the NEP activity was purchased from Bachem Biosciences, Germany. IKK inhibitor (wedelolactone), NFκB inhibitor (BAY 11-7082), and rhTNFα were all from Sigma Aldrich, UK. Recombinant human NEP enzyme (rhNEP) was a kind offer by Dr David Nanus, Weill Cornell Medical College, New York, USA. Proteasome inhibitor (Bortezomib, VELCADE) was purchased from Janssen-Cilag Pharmaceuticals, Greece. 20S proteasome control and proteasome inhibitor lactacystin were from Chemicon International, USA. Protein quantification was done with the use of the Bradford quantification assay (Bio-Rad Laboratories, Inc.) for the total cell lysates, and the BCA Protein Kit (PIERCE Endogen, UK) for the nuclear and cytoplasmic extracts.

### Total cell lysates

Total protein cell lysates were prepared using a 0.5% CHAPS buffer, which did not affect NEP and proteasomal enzymatic activity. Total lysates were also prepared using a second buffer (containing 10 mM Tris-HCl, 50 mM EDTA, 150 mM NaCl, 1% Triton-X and 10% Glycerol) for western blotting purposes.

### Nuclear extracts

Nuclear extracts were prepared as described by Carter *et al. *[18], with minor modifications as reported previously [19]. 10^6 ^cells were washed in cold PBS and collected in 400 μl of ice-cold lysis buffer supplemented with 10 μg/ml of protease inhibitors cocktail and then incubated on ice for 20 min. Nonidet (NP-40) 10% was added to lyse the cells which were vortexed and centrifuged for 20 sec at 4°C at 13,000 rpm. The supernatant containing the cytoplasmic extracts stored at -80°C. The pellet was resuspended in 100 μl of extraction buffer for 20 min on ice. The nuclear suspension was then centrifuged for 15 minutes at 13,000 rpm and supernatant nuclear extracts stored at -80°C until use. Nuclear extracts of PC-3 cells were prepared at baseline and following bortezomib incubation at a dose of 1 μM for 60 min.

### Flow cytometry analysis

Cells were gently removed from tissue culture flasks with the use of a cell scraper, rinsed twice in PBS and resuspended in culture medium and analysed for CD10 surface expression using a fluorescein-conjugated mouse monoclonal antibody against CD10 (clone ALB1, Beckman Coulter). A mouse anti-human IgG1-FITC antibody (Beckman Coulter) was used as isotype control. Analysis was performed with an Argon and HeNe laser flow cytometer (Beckman Coulter).

### NEP enzymatic activity assay

Chromogenic NEP activity assay in total cell lysates was performed as described before [14]. Absorbance was read at 540 nm using a Wallac Victor™ multilabel counter. All experiments were performed in quadruplicate. Specific activity at baseline was calculated as picomoles of converted substrate per microgram protein per minute. Values were compared against a substrate-specific standard curve and an rhNEP enzyme activity curve.

### ET-1 ELISA

Culture supernatants were collected when cells were at 80% confluency. A commercially available kit based on a double-sandwich assay specific for ET-1 detection was used (IBL Internaitonal). Absorbance was read at 450 nm according to kit specifications with the use of a multilabel counter as above. ET-1 concentration in the cell culture supernatant was estimated in pg/ml based on absorbance unit plot readings.

### 20S proteasome activity assay

Chymotryptic activity of the 20S proteasome was measured in total cell lysates with the use of a commercially available fluorogenic assay kit based on 7-Amino-4-methylcoumarin (AMC) (Chemicon International, USA) and confirmed with in-house developed assays. All experiments were performed in quadruplicate and measured using a Wallac Victor™ multilabel counter with 380 nm excitation and 490 nm emission wavelengths. Fluorometric reading of baseline enzyme activity was expressed as RFU/μg of total protein. Values were compared against a fluorogenic substrate (LLVY-AMC) standard curve and a 20S proteasome control activity curve. Changes in proteasomal activity following different drug-incubations were expressed as percentage increase or decrease from baseline activity of each line. Bortezomib incubations were at a dose of 1 μM for 60 minutes in LnCaP and PC-3 cells. Percentage of activity inhibition of control 20S proteasome was produced by lactacystin. All measurements were performed in triplicate (*p *< 0.001).

### Western blot analysis

30 μg of total protein lysate of each sample (total cell lysate or nuclear/cytoplasmic extracts) were loaded on 4-12% Bis-Tris polyacrylamide gels and underwent electrophoresis under reducing conditions. Proteins were subsequently transferred on a PVDF blotting membrane. Following blocking with 5% non-fat milk, membranes with incubated with primary antibodies at 4°C overnight. Primary antibodies against CD10 (clone 56C6, mouse monoclonal, Novocastra), the p65 subunit of NFκB (F-6, mouse monoclonal and C-20, goat polyclonal, Santa Cruz Biotechnology, Inc.), IκBα (C-15, rabbit polyclonal, Santa Cruz Biotecnhology, Inc.) and actin (clone AC-40, mouse monoclonal, Sigma Aldrich, UK) were used. Secondary antibodies incubations were at a dilution of 1:2500 for 2 hours at room temperature. Both colorimetric (Opti4CN Detection Kit, Bio-Rad Laboratories, Inc) and chemiluminescence detection (ECL detection reagent, Amersham Biosciences) with autoradiography were used. Total cellular IκBα levels in PC-3 cells were prepared following serial incubations with bortezomib at a dose of 1 μM for 30, 45, 60 and 90 min respectively.

### Immunocytochemistry

Cells were spread and cultured on glass slides. When at 80-90% confluency cells were fixed with Merckofix^® ^spray fixative (Merck KGaA, Darmstadt, Germany) and conventional avidin-biotin immunocytochemistry was performed. The Ventana NexES Automated Slide Stainer and related Ventana reagents were used. The samples were immersed in a citrate buffer solution (pH 7.3) and heated for 15 min at 350 W. They were subsequently incubated with 3% H_2_O_2 _for 4 min to quench the endogenous peroxidase activity. A primary antibody against the p65 subunit of NFκB was used in a 1:100 dilution. Diaminobenzidine (DAB) was used as a chromogen for detection of the antigens. Incubation with copper sulfate was performed for enhancement of the colour reaction. The slides were finally counterstained with haematoxylin and coverslipped for examination. Experimental conditions involved drug-incubation of PC-3 cells with bortezomib at a dose of 1 μM for 60 min, wedelolactone at a dose of 50 μM for 90 min and Bay-7082 at a dose of 20 μM for 16 hrs. PC-3 cells were incubated with rhNEP at a dose of 50 μg/ml for 72 hrs.

### Electrophoretic mobility shift assay

As NFκB consensus oligonuclotide we used the PRDII element [20] of human IFN-beta enhancer (5'-TGGCCAACATGGTGAAACCCCGTTTCTACT-3') (IMBB, Microchemistry Laboratory), labelled with [γ-^32^P] ATP using T4 polynucleotide kinase. The probe was purified through G50 columns (Amersham Biosciences) and then EMSA was performed. Briefly, 3 μg of nuclear extracts were incubated at RT for 20 min with 100 ng of labelled double-stranded oligonucleotide in the presence of 20 ng of PolydI-dC (PIERCE Endogen, UK) and 20 μg of BSA. Nuclear extracts from HeLa cells (ATCC, UK) 6 hours post-infection with the Sendai paramyxovirus (Cantell strain) and following incubation with TNFα for 1 hour were used as positive controls. For the supershift assay, nuclear extracts were incubated with 1 μg of anti-p65 rabbit polyclonal antibody (Santa Cruz Biotechnology, Inc.) for 30 min at 4°C, before the addition of the probe. In every case, the protein-DNA complexes were separated on a 7% non-denaturing polyacrylamide gel and bands were visualized using a Taeffun Phospor-Imager/Scanner with the ImageQuant™ TL analysis software (Amersham Biosciences).

## Results

### NEP/NPs axis at steady state

**Figure 1:**
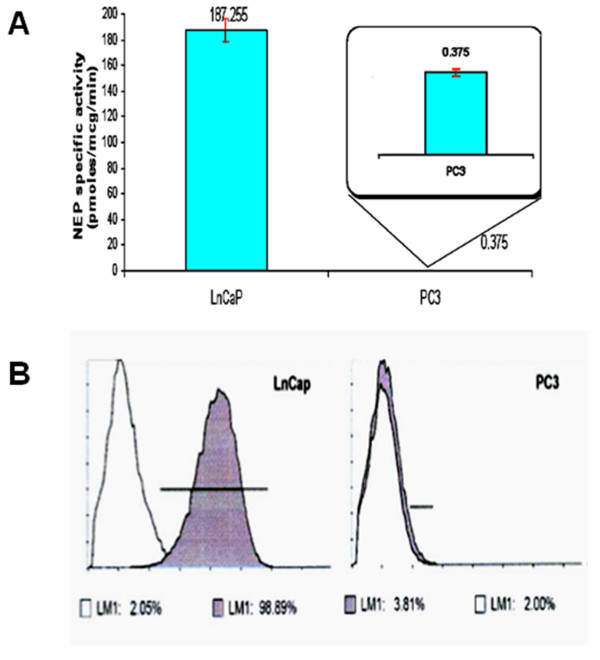
**NEP surface expression and activity in PC cell lines**. (A) NEP specific activity (pmoles/μg/min) in LnCaP (*left*; 187.255, SD: 2.84) and PC-3 cells (*right*; 0.375, SD: 0.03). All measurements in quintuplicate (*p < 0.001*). (B) Flow cytometry analysis of NEP surface expression in LnCaP (*left*) and PC-3 cells (*right*). All measurements in triplicate (*p < 0.001*).

Baseline surface NEP expression in LnCaP cells was estimated to be at a 99% level (MFI = 30). NEP enzymatic activity was 187 pmoles of converted substrate per microgram of protein per minute. PC-3 cells were negative for NEP expression on flow cytometry analysis, and showed negligible NEP enzymatic activity (0.375 pmoles/μg/minute) (Figures 1A, B).

**Figure 2:**
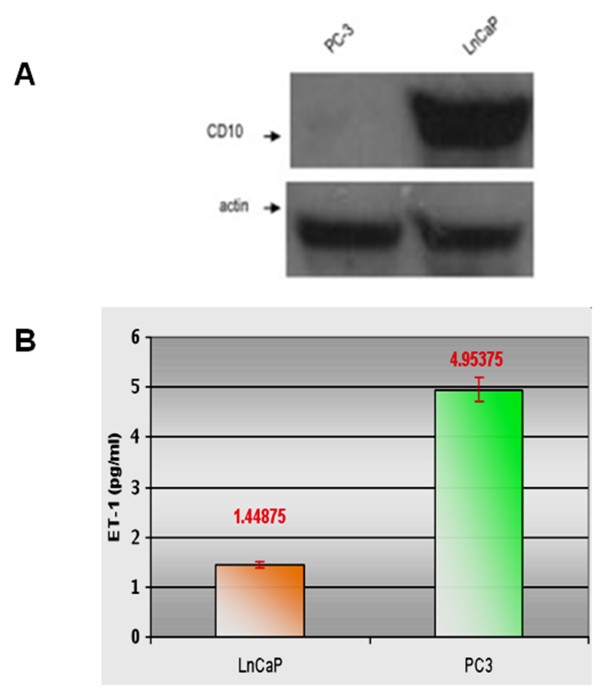
**NEP cellular expression and ET-1 secretion in PC cell lines**. (A) Total cellular NEP levels in PC-3 (*left*) and LnCaP cells (*right*). (B) ET-1 ELISA detection in culture supernatants of LnCaP (*left*; 1.448, SD: 0.06) and PC-3 cells (*right*; 4.953, SD: 0.49). All measurements in octuplicate (*p < 0.001*).

The estimation of NEP protein amount in whole cell lysates via western blot concurred with the above, showing bands of increased intensity for LnCaP cells and no bands for PC-3 cells (Figure 2A). Secreted ET-1 concentration measure in cell culture supernatants was 1.45 pg/ml in the LnCaP line and 4.95 pg/mg in the PC-3 line (Figure 2B).

### NFκB/UPS pathway at steady state

**Figure 3:**
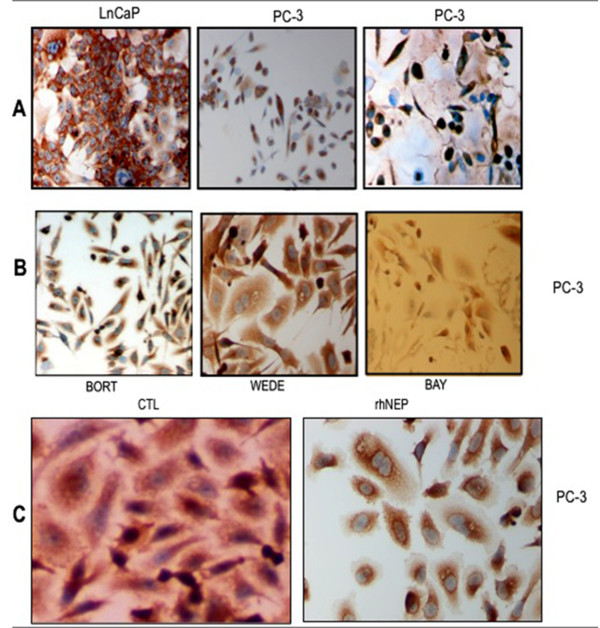
**NFκB immunohistochemical expression and subcellular localisation in PC cell lines**. (A) LnCaP cells (*left*, ×10) and PC-3 cells (*middle and < right*, ×10 and ×40). (B) PC-3 cells; incubations with: *Left*: bortezomib 1 μM (60 min) (×10); *Middle*: wedelolactone 50 μM (90 min) (×40); *Right*: Bay-7082 20 μM (16 hrs) (×10). (C) PC-3 cells; *Left*: Baseline (×40); *Right*: Incubation with rhNEP 50 μg/ml (72 hrs) (×40).

On immunocytochemistry analysis, LnCaP cells showed almost exclusively cytoplasmic NFκB localisation, while PC-3 cells showed mixed nuclear and cytoplasmic localisation (combination of cytoplasmic, mixed or purely nuclear localisation) (Figure 3A).

As expected, use of UPS inhibitors (NFκB, IKK and 20S proteasome inhibitor) converted the mixed NFκB localisation in PC-3 cells to cytoplasmic (Figure 3B). Higher concentration or longer incubation with two of these inhibitors (NFκB inhibitor BAY-7082 50 μM for 16 hours; IKK inhibitor wedelolactone 50 μM for 5 hours) resulted in altered cell morphology (round, plump, apoptotic-looking cells) (data not shown). Use of UPS inhibitors expectedly had no effect on NFκB localisation in LnCaP cells, which remained cytoplasmic. When PC-3 cells were incubated with rhNEP, their baseline NFκB sub-cellular localization profile changed from mixed nuclear and cytoplasmic to purely cytoplasmic (Figure 3C).

**Figure 4:**
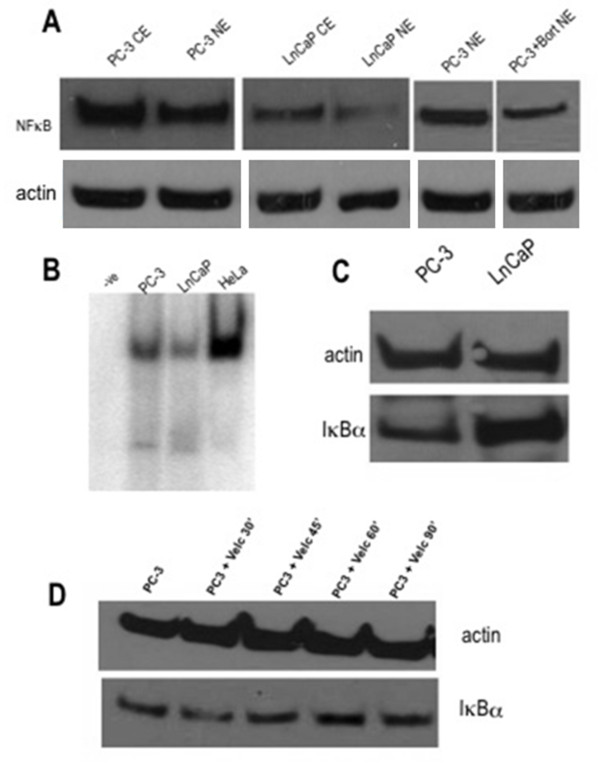
**NFκB activation in PC cell lines**. (A) Nuclear and cytoplasmic NFκB levels in PC-3 (*left*) and LnCaP cells (*middle*). *Right*: Nuclear extracts of PC-3 cells at baseline and following bortezomib incubation (1 μM for 60 min). (B) NFκB EMSA. *Lane 1*: negative control; *Lane 2*: PC-3 cells; *Lane 3*: LnCaP cells; *Lane 4*: HeLa cells (6 hrs post-infection). (C) Total cellular IκBα levels in PC-3 (*left*) and LnCaP cells (*right*). (D) Total cellular IκBα levels in PC-3 cells following serial incubations with bortezomib 1 μM.

Western blot analysis of nuclear and cytoplasmic extracts in LnCaP cells showed less nuclear NFκB protein and less amount of cytoplasmic protein compared to PC-3 cells, which showed significant amount of both nuclear and cytoplasmic NFκB protein. Bortezomib incubation predictably resulted in reduced nuclear NFκB amount in PC-3 cells (Figure 4A). The use of a more sensitive technique such as EMSA did detect NFκB-DNA binding signal in LnCaP cells, however less intense compared to PC-3 cells (Figure 4B).

Furthermore, LnCaP cells also showed significantly higher amount of total IκBα protein compared to PC-3 cells (Figure 4C). The effect of the proteasome inhibitor on the IκBα status, much evidenced in the literature, was also demonstrated on serial incubations of bortezomib alone in PC-3 cells. There was a gradual time-dependent increase in the total IκBα amount, peaking at the 60-minute incubation, and declining at longer incubation, consistent with the reversible nature of bortezomib-induced proteasome inhibition [21] (Figure 4D).

**Figure 5:**
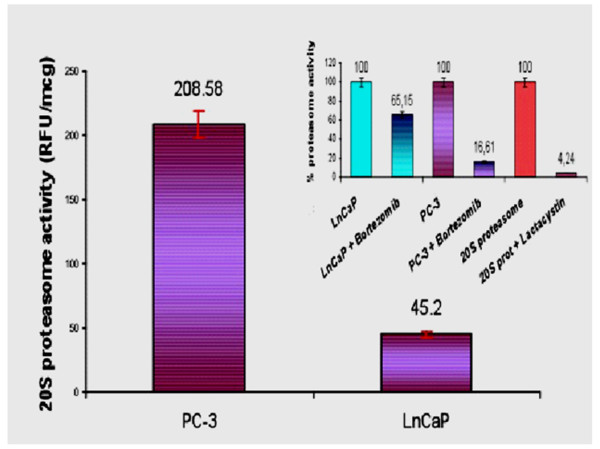
**Proteasome activity in PC cell lines**. 20S proteasome activity (RFU/μg) in PC-3 (*left*; 208.58, SD: 3.24) and LnCaP cells (*right*; 45.2, SD: 0.2). All measurements in quintuplicate (*p < 0.001*). *Small graph*: % inhibition of baseline 20S proteasome inhibition produced by bortezomib incubations (1 μM for 60 minutes) in LnCaP (*left*) and PC-3 cells (*middle*); % activity inhibition of control 20S proteasome produced by lactacystin (*right*). All measurements in triplicate (*p < 0.001*).

20S proteasomal activity measurement showed the opposite relationship between LnCaP and PC-3 cells as compared to NEP activity. LnCaP showed low proteasomal activity (45.2 RFU/μg protein), while PC-3 cells had a significantly higher activity (208.6 RFU/μg protein; *p *< 0.001). Incubation with proteasome inhibitor bortezomib produced a significant proteasomal inhibition in PC-3 cells (17% of baseline activity) and a moderate one in LnCaP cells (65% of baseline activity). The inhibition in PC-3 cells was comparable with the inhibition produced by lactacystin on 20S proteasome control protein (Figure 5).

Our results are suggestive of a mirror-image profile of the NEP/NPs and NFκB/UPS pathways between AD and AI PC cells (Table 1).

**Table 1:** In vitro profile of the NEP/NPs and NFκB/UPS pathways in PC

	***AD***	***AI***
	***LnCaP***	***PC-3***
**NEP specific activity (pmoles/μg/min)**	187.25	0.375
**NEP surface expression (%)**	98.89	3.81
**NEP protein**	High	None
**ET-1 secretion (pg/ml)**	1.44	4.95
**NFκB subcellular localisation**	Cytoplasmic	Mixed
**Nuclear NFκB protein**	Low	High
**NFκB-DNA binding signal**	Low	Intense
**Total cellular IκBα protein**	High	Low
**20S proteasomal activity (RFU/μg)**	45.2	208.58

Analysis of components of the two pathways in cell line models of AD and AI states.

## Discussion

The importance of NPs in the emergence of castration-resistant PC clones with metastatic potential has attracted a renewal of interest in the recent years, supplemented by our modern knowledge of complex tumour-niche interactions. Both ET-1 and bombesin have been shown to activate pathways and processes that promote tumour invasion and metastasis in the microenvironment of PC [22-26].

Emerging preclinical evidence implicates NFκB/UPS pathway in the development, growth, survival, angiogenesis and metastatic progression of PC cell lines and preclinical models [27,28]. NFκB has been shown to be constitutively active in PC cell lines, preventing apoptotic cell death [29,30]. Constitutive activation of NFκB has also been detected in AI PC xenografts and in PC tissues [15,29-31].

Our results provide evidence that at baseline level there is an inverse pattern of expression between certain components of the NFκB/UPS and the NEP/NPs pathways. As described above, these two pathways have been previously separately implicated in PC and the progress towards castration resistance. We have now demonstrated that LnCaP cells, modelling the AD state, which are known to express NEP and therefore cleave endogenous/paracrine NPs, also exhibit low proteasomal activity. This translates to low I B degradation rate and resultant high total I B levels. As such, these cells have constitutively low level of NFκB activation, indicated by its cytoplasmic localisation and low DNA-binding signal.

PC-3 cells, modelling progression to castration resistance, have lost their NEP expression [14] due to promoter methylation [32] and therefore have significant levels of autocrine and paracrine-acting NPs available for cellular signaling, as our results establish. We have also demonstrated that they exhibit high proteasomal activity, resulting in low I B levels and increased NFκB activation. PC-3 cells appreciably showed a greater sensitivity to proteasomal inhibition compared to LnCaP cells. The biological explanation behind this might be that there is a minimal requirement of 20S proteasomal activity for cellular homeostasis, which cannot be abolished. As such, PC-3 cells are amenable to a high percentage inhibition of proteasomal activity exactly because they have higher baseline activity, while LnCaP cells, having a lower baseline activity, are "allowed" a smaller-scale inhibition.

It is therefore possible that, in the progression to androgen-independence, cells via loss of their membrane NEP activity, as shown before [14], survive and proliferate in a milieu of increased paracrine NP signaling. Indeed, our results relating to the AD state *in vitro *model (represented by LnCaP cells) support that the latter have lower secreted levels of ET-1, also shown by Nelson *et al. *[13] and Grant *et al. *[33], which could be the combined outcome of both NEP-mediated degradation but also reduced ET-1 production due to decreased ECE-1 expression [34]. Concomitantly, the AI *in vitro *model (represented by PC-3 cells) exhibits activation of the NFκB signaling pathway with up-regulation of their baseline cellular proteasomal activity.

## Conclusions

Our study provides evidence of a molecular pattern between the NEP/NPs and NFκB/UPS pathways that is inverted in the progression from hormone-naïve to hormone-refractory PC. This biological concept can be used towards combined clinical applications of antagonists/inhibitors involving these two mechanisms, possibly in conjunction with standard chemotherapy, thus more effectively blocking pathways know to affect survival of PC.

## Abbreviations

AD: androgen-dependent; AI: androgen-independent; AMC: 7-Amino-4-methylcoumarin; BCA: bicinchoninic acid; BSA: bovine serum albumin; ECACC: European collection of cell cultures; ECE-1: endothelin converting enzyme 1; ECL: enhanced chemiluminescence; EMSA: electrophoretic mobility assay shift; FBS: fetal bovine serum; ET-1: endothelin-1; IFN: interferon; IκB: inhibitor kappa B; IKK: IκB kinase; IL-6: interleukin 6; NEP: neutral endopeptidase, CD10; NFκB: nuclear factor kappa B; NP: neuropeptide; PBS: phosphate buffer saline; PC: prostate cancer; PSA: prostate-specific antigen; PVDF: polyvinylidene fluoride; RFU: relative fluorescence units; rh: recombinant human; TNFα: tumor necrosis factor α; UPS: ubiquitin-proteasome system.

## Competing interests

The authors declare that they have no competing interests.

## Authors' contributions

CNP and AP designed the study. IAV, EH, RMV, CD and EA performed experiments. AP and PJV drafted the manuscript. PJV and CNP revised the manuscript. All authors read and approved the final manuscript.
